# Rates of Attrition and Dropout in App-Based Interventions for Chronic Disease: Systematic Review and Meta-Analysis

**DOI:** 10.2196/20283

**Published:** 2020-09-29

**Authors:** Gideon Meyerowitz-Katz, Sumathy Ravi, Leonard Arnolda, Xiaoqi Feng, Glen Maberly, Thomas Astell-Burt

**Affiliations:** 1 Western Sydney Diabetes Western Sydney Local Health District Blacktown NSW Australia; 2 School of Health and Society University of Wollongong Wollongong Australia; 3 Illawarra Health & Medical Research Institute Wollongong Australia; 4 School of Public Health and Community Medicine University of New South Wales Kingsford NSW Australia; 5 Menzies Centre for Health Policy University of Sydney Camperdown NSW Australia

**Keywords:** chronic disease, mHealth, mobile apps, attrition, dropout

## Abstract

**Background:**

Chronic disease represents a large and growing burden to the health care system worldwide. One method of managing this burden is the use of app-based interventions; however attrition, defined as lack of patient use of the intervention, is an issue for these interventions. While many apps have been developed, there is some evidence that they have significant issues with sustained use, with up to 98% of people only using the app for a short time before dropping out and/or dropping use down to the point where the app is no longer effective at helping to manage disease.

**Objective:**

Our objectives are to systematically appraise and perform a meta-analysis on dropout rates in apps for chronic disease and to qualitatively synthesize possible reasons for these dropout rates that could be addressed in future interventions.

**Methods:**

MEDLINE (Medical Literature Analysis and Retrieval System Online), PubMed, Cochrane CENTRAL (Central Register of Controlled Trials), and Embase were searched from 2003 to the present to look at mobile health (mHealth) and attrition or dropout. Studies, either randomized controlled trials (RCTs) or observational trials, looking at chronic disease with measures of dropout were included. Meta-analysis of attrition rates was conducted in Stata, version 15.1 (StataCorp LLC). Included studies were also qualitatively synthesized to examine reasons for dropout and avenues for future research.

**Results:**

Of 833 studies identified in the literature search, 17 were included in the review and meta-analysis. Out of 17 studies, 9 (53%) were RCTs and 8 (47%) were observational trials, with both types covering a range of chronic diseases. The pooled dropout rate was 43% (95% CI 29-57), with observational studies having a higher dropout rate (49%, 95% CI 27-70) than RCTs in more controlled scenarios, which only had a 40% dropout rate (95% CI 16-63). The studies were extremely varied, which is represented statistically in the high degree of heterogeneity (I^2^>99%). Qualitative synthesis revealed a range of reasons relating to attrition from app-based interventions, including social, demographic, and behavioral factors that could be addressed.

**Conclusions:**

Dropout rates in mHealth interventions are high, but possible areas to minimize attrition exist. Reducing dropout rates will make these apps more effective for disease management in the long term.

**Trial Registration:**

International Prospective Register of Systematic Reviews (PROSPERO) CRD42019128737; https://www.crd.york.ac.uk/prospero/display_record.php?ID=CRD42019128737

## Introduction

Chronic diseases are a large and growing issue worldwide, with rates increasing dramatically in recent years, including infectious diseases that are now managed chronically, such as HIV. One example is diabetes, with global prevalence nearly doubling from less than 5% in the 1990s to more than 8% today [1]. As with other chronic diseases, the economic and social cost of diabetes is enormous, with large direct health care costs often eclipsed by the societal impacts of the disease [1,2]. This has led to a large body of research focusing on how to prevent and manage these diseases, with many recommendations now advising moving from a model of care that is medically focused to patient-centric and community-focused care [3]. However, there are many difficulties in implementing programs for chronic disease prevention and care, in particular, the challenges posed by catering to a large, diverse, and growing population of people requiring these services [1,4]. One such difficulty is the dropout rate, or attrition, whereby patients discontinue use of interventions either entirely or enough that the benefit from the intervention is negligible. This is an area of particular concern for new technological innovations, such as mobile phone apps.

The management of chronic disease is often complex. Patients may be on numerous medications, follow strict dietary regimens, and have lifestyle goals to fulfil to optimally manage their disease [5]. Professional assistance from health workers—doctors, educators, dieticians, and others—is an important component of this management, but increasingly, international evidence has shown that self-management—empowering patients to manage their own care—is another effective way to improve outcomes [6-8].

Self-management interventions range from providing educational materials to highly supportive, multifaceted programs that include a variety of measures [7]. One method of self-management assistance that is increasingly popular is providing web-based eHealth or mobile apps (ie, mobile health [mHealth]) to people in order to assist in their management of their disease [9]. These interventions have demonstrated efficacy in terms of markers for management, with a recent systematic review finding that, although the evidence is preliminary, mHealth interventions are effective in reducing weight and glycated hemoglobin in people with diabetes [10]. Another recent review looking only at the efficacy of mobile apps for diabetes care found that there was limited evidence supporting the effectiveness of diabetes apps to improve blood glucose markers for people with diabetes [11]. Overall, there is a growing body of evidence that mHealth interventions, and apps in particular, may be an effective method of promoting self-management in patients.

However, a major barrier to patient care in the use of mHealth interventions is attrition. Previous research has identified that up to 80% of all participants in mHealth interventions may engage in only *minimal use* of these interventions, defined as logging in to the service less than twice, and only a small fraction of users consistently use the intervention long term [12,13]. While clinical trials often report 70% or higher retention, these are often short in duration, some fewer than two months, and may not represent the situation in real-world use [10]. One observational trial of app usage in a large real-world cohort found that only 2% had sustained continuous use of the kind that would be expected to improve clinical outcomes [14]. If only 2% of people who download an app actually use it, there is clearly minimal benefit for the majority. Demonstrating that mHealth interventions are effective in clinical trials is not enough: retention in real-world settings is a necessary precondition for these interventions to be considered effective.

This paper presents a systematic review and meta-analysis into the rate and causes of dropout in mHealth interventions for diabetes and other chronic health issues. This is divided into clinical trials and observational research studies in order to estimate the rates in both controlled and uncontrolled settings and to estimate the effect both in studies with a large support network to prevent attrition and in the more *real-world* experience that might be expected when these apps are actually rolled out into clinical practice. These were also qualitatively synthesized.

## Methods

A reproducible strategy was used to identify studies examining mHealth interventions for self-management of chronic disease, either mobile app based or internet based. Studies were identified by electronically searching MEDLINE (Medical Literature Analysis and Retrieval System Online), PubMed, Cochrane CENTRAL (Central Register of Controlled Trials), and Embase from 2003 to the present day. Search terms are fully outlined below and are loosely based on previous systematic reviews looking at similar topics [15]. Searches were performed in June 2019 by GMK, and duplicates were excluded using Microsoft Excel 2013 and EndNote, version 8.0 (Clarivate).

Electronic downloads of searched titles were then performed using the data collection process for each individual database, with titles being screened by GMK and SR against inclusion criteria to determine eligibility. Abstracts were then reviewed by these two reviewers independently. Any disagreements were adjudicated between the two authors. References from included studies were also assessed to identify further trials for inclusion. Both experimental and quasi-experimental study designs were included in this review. As the analysis is based on a secondary endpoint (ie, attrition), no formal risk-of-bias tool was used to assess the quality of included studies.

For the meta-analysis, the total rate of dropout was extracted from each study, as well as the number of participants in the control and intervention groups. The primary summary measure was the rate of dropout in these trials.

Eligibility criteria for inclusion of studies are as follows—studies must meet all criteria:

1. Published in English.
2. Addressed to an adult population (≥18 years of age).
3. Either randomized controlled trials (RCTs) or observational interventions (ie, case control or cohort).
4. Look at app usage in chronic disease.
5. Include a measure of dropout and attrition.

A systematic narrative synthesis was produced to describe the included studies and their findings relating to dropout. This narrative synthesis reviewed the findings from all included studies and provided an overall summation of the subject matter.

Stata, version 15.1 (StataCorp LLC), was used to perform the meta-analysis of the included studies, using the *metaprop* command, with results pooled from RCTs looking at the rate of dropout in clinical trials. There was also a second meta-analysis, by trial type: observational versus RCT. The primary outcome was the rate of dropout. Heterogeneity was assessed using the I^2^ statistic and visual inspection of funnel plots; the Egger weighted meta-regression test was used to determine the influence of publication bias. If studies are identified that attempted to prevent dropout, these will form the basis of a subgroup analysis. A sensitivity analysis was conducted looking at attrition comparing short (≤2 months) studies with long (>2 months) studies.

This study was registered at the International Prospective Register of Systematic Reviews (PROSPERO) (CRD42019128737).

## Results

### Overview

Use of mHealth solutions in managing chronic conditions is increasing; however, the effective and long-term engagement (ie, attrition rate) has been attributed to various factors.

**Figure 1:**
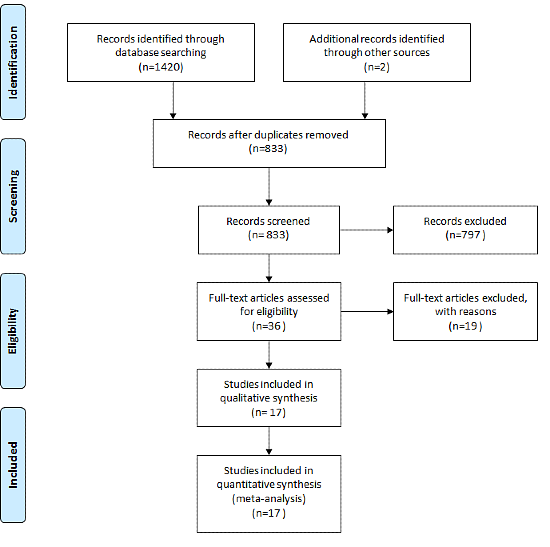
PRISMA (Preferred Reporting Items for Systematic Reviews and Meta-Analyses) flow diagram.

After database searches were performed, a total of 1420 articles were identified. After excluding duplicates, 831 unique records remained. Of these, 797 were excluded prior to review. A further 2 records were identified through reference screening from included studies, leaving a total of 36 studies to be included in the review (see Figure 1). Of the 36 studies included in the final review, 19 were excluded based on the exclusion criteria of studies including children and studies that only looked at acute or infectious diseases. Studies that were purely online, telephone and texting interventions, and studies that did not have any measurement of rates of dropout and attrition were also excluded. This left 17 studies to be included in the final qualitative and quantitative synthesis.

### Characteristics of Included Studies

Included studies were published between 2011 and 2019 [14,16-31]. Of these, most (14/17, 82%) examined a range of chronic diseases, including single studies targeting lower back pain, chronic kidney disease, pain, dysmenorrhea, and HIV medications; the remainder (9/17, 53%) looked at more general lifestyle improvement, such as eating behavior and physical activity. Out of 17 studies, 3 (18%) that were included in the review looked specifically at diabetes. There were 9 RCTs (53%) included in the final synthesis and 8 observational trials (47%). These are summarized in Table 1.

Studies ranged significantly in duration, size, attrition rate, methodology, and other areas. The shortest trial included in this review lasted 2 weeks, and a total of 5 out of 17 (29%) lasted one month or less. Out of 9 RCTs, 2 (22%) looked at 1 year of data, and a number of observational trials were conducted over a period of 6-10 months. The lowest attrition rate in any study was 9% in an RCT lasting 1 year [29]; the highest attrition rate was 82% in an observational trial lasting 6 weeks [26]. The largest trial was an observational study lasting 24 weeks, with nearly 200,000 participants; the smallest trial was a small cohort study including just 20 people.

**Table 1:** Summary of studies.

Author	Name of the study	Year published	No. of participants	Area of study	Attrition rate	Type of study
Cook et al, [16]	A Counselor in Your Pocket: Feasibility of Mobile Health Tailored Messages to Support HIV Medication Adherence	2015	37	HIV medication adherence	60%	Observational study
Torbjørnsen et al, [17]	A Low-Intensity Mobile Health Intervention With and Without Health Counseling for Persons With Type 2 Diabetes, Part 1: Baseline and Short-Term Results From a Randomized Controlled Trial in the Norwegian Part of RENEWING HEALTH	2014	151	Self-management support for people with type 2 diabetes	18%	RCT^a^
Elbert et al, [18]	A Mobile Phone App Intervention Targeting Fruit and Vegetable Consumption: The Efficacy of Textual and Auditory Tailored Health Information Tested in a Randomized Controlled Trial	2016	342	Fruit and vegetable consumption	55%	RCT
Selter et al, [19]	An mHealth App for Self-Management of Chronic Lower Back Pain (Limbr): Pilot Study	2018	93	Self-management of chronic lower back pain	62%	Pilot study
Lee et al, [20]	Effect of Self-Monitoring on Long-Term Patient Engagement With Mobile Health Applications	2018	1439	Self-monitoring health app	46%	Observational study
Chen et al, [21]	Effects of Journaling Dietary Intake App on the Health Outcomes of Chronic Kidney Disease Stage 3B-5	2016	20	Chronic kidney disease	35%	Observational study
Mak et al, [22]	Efficacy and Moderation of Mobile App-Based Programs for Mindfulness-Based Training, Self-Compassion Training, and Cognitive Behavioral Psychoeducation on Mental Health: Randomized Controlled Noninferiority Trial	2018	2161	Mental well-being	76.5% and 83.9%	RCT
Guertler et al, [23]	Engagement and Nonusage Attrition With a Free Physical Activity Promotion Program: The Case of 10,000 Steps Australia	2015	16,948	Physical activity promotion: 10,000 steps	25% to 75%	Observational study
Helander et al, [14]	Factors Related to Sustained Use of a Free Mobile App for Dietary Self-Monitoring With Photography and Peer Feedback: Retrospective Cohort Study	2014	189,770	Diet self-monitoring	86.39%	Retrospective cohort study
Fukuoka et al, [24]	Identifying Factors Associated With Dropout During Prerandomization Run-in Period From an mHealth Physical Activity Education Study: The mPED Trial	2015	318	Physical activity education	34%	Observational study
Spring et al, [25]	Multicomponent mHealth Intervention for Large, Sustained Change in Multiple Diet and Activity Risk Behaviors: The Make Better Choices 2 Randomized Controlled Trial	2018	212	Diet behavior	17.90%	RCT
Roepke et al, [26]	Randomized Controlled Trial of SuperBetter, a Smartphone-Based/Internet-Based Self-Help Tool to Reduce Depressive Symptoms	2015	283	Reduce depressive symptoms	81.66%	RCT
Druce et al, [31]	Recruitment and Ongoing Engagement in a UK Smartphone Study Examining the Association Between Weather and Pain: Cohort Study	2017	6370	Weather and pain	N/A^b^	Observational study
Hales et al, [27]	Social Networks for Improving Healthy Weight Loss Behaviors for Overweight and Obese Adults: A Randomized Clinical Trial of the Social Pounds Off Digitally (Social POD) Mobile App	2016	51	Weight loss behavior	12%	RCT
Blödt et al, [28]	Effectiveness of App-Based Self-Acupressure for Women With Menstrual Pain Compared to Usual Care: A Randomized Pragmatic Trial	2018	221	App-based self-acupressure for menstrual pain	14%	RCT
Karhula et al, [29]	Telemonitoring and Mobile Phone-Based Health Coaching Among Finnish Diabetic and Heart Disease Patients: Randomized Controlled Trial	2015	517	Health coaching: diabetes and heart disease patients	8.90%	RCT
Holmen et al, [30]	A Mobile Health Intervention for Self-Management and Lifestyle Change for Persons With Type 2 Diabetes, Part 2: One-Year Results From the Norwegian Randomized Controlled Trial RENEWING HEALTH	2014	151	Self-management support for people with type 2 diabetes	21%	RCT

^a^RCT: randomized controlled trial.

^b^N/A: not applicable; this value was not reported.

### Meta-Analysis

**Figure 2:**
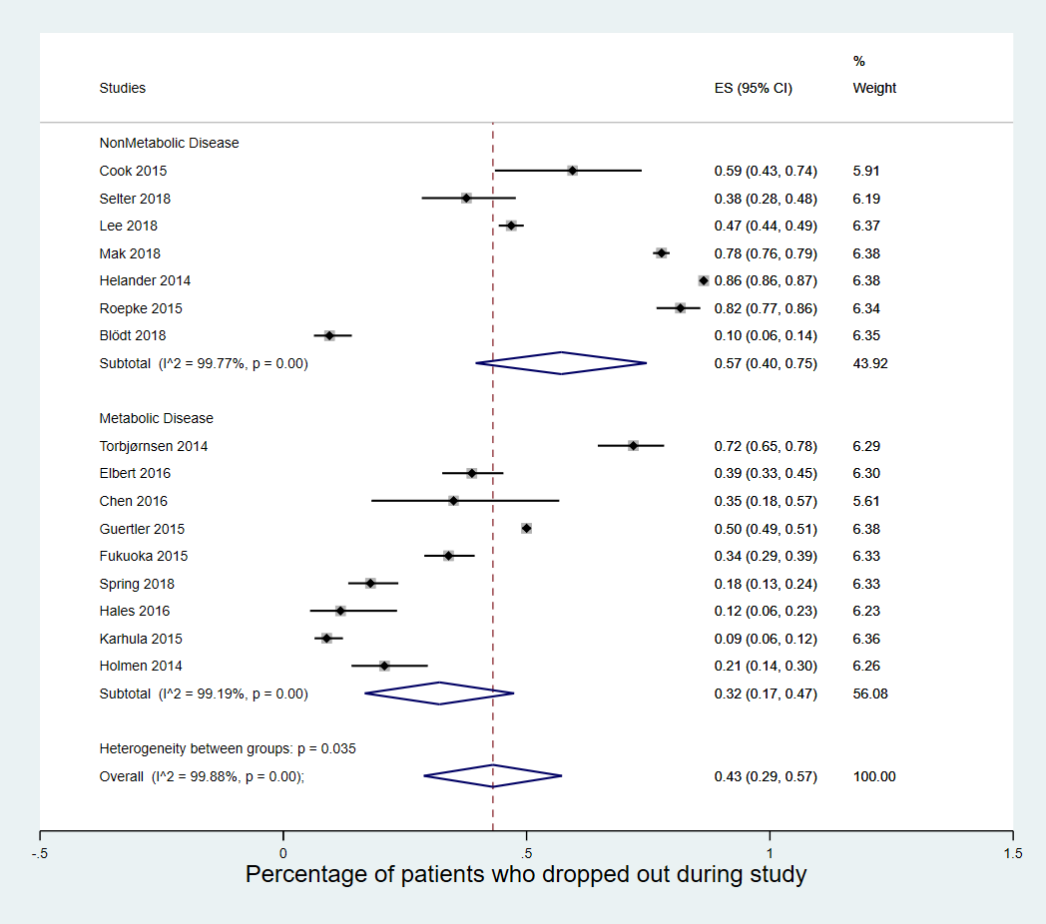
Meta-analysis of attrition rates in app-based intervention studies for chronic disease. ES: effect size.

Results from the meta-analysis are presented in Figure 2. The average attrition rate overall was 43% (95% CI 29-57), with very high between-study heterogeneity indicated by an I^2^ statistic of >99%. The very high heterogeneity is not unexpected in this case, as studies were extremely varied in terms of time, implementation, and the disease state that they were examining.

Looking at the breakdown of results by the type of study, there was a higher degree of attrition in the observational *real-world* studies (49%, 95% CI 27-70) than in the RCTs in more controlled scenarios, with only 40% (95% CI 16-63) dropping out. Sensitivity analyses looking at differences in length of study (ie, short vs long), diabetes versus other chronic diseases, or whether the studies were numerically large did not find any similar differences in attrition rate between trials.

### Attrition Rates

One reason associated with lower attrition rates was the behavioral characteristics of the included participants. Low attrition rates were characterized by reasons such as the perception of own health as poor—thus, incentivizing the need to change [18]—and those who wanted to be involved in their health care [20]. Other factors that were associated with attrition in included studies were health literacy; age, with younger participants dropping out less; and postgraduate education [18,22]. Very low attrition was also reported among those who were on strict diets or who had been healthy eaters prior to the initiation of the study [14]. Another association with low attrition was with those engaged in multiple interventions. Those engaged in internet or phone programs as well as apps were more likely to remain in the research study [23]. Conversely, there did not appear to be much influence on attrition rates in terms of length of study, the disease studied, or the size of the app trial.

Findings from these studies suggest ways to improve attrition rate and long-term engagement by using varying message contents or formats to maintain users’ interests; for example, tailored messages may have the potential to improve adherence to a clinically significant degree [16]. Other studies suggest less of a benefit from tailoring messages to maintain users’ interest; despite a low attrition rate of 22% at 4 months and 1 year in two studies, respectively, an app and health counseling did not reduce hemoglobin A1c levels between the intervention and usual care groups [17,30]. In addition, self-management skills and the ability to contact health professionals were found to increase engagement, while users’ feedback input improved usability of apps and enhanced user experiences for daily self-reports [17,19]. Classifying different types of users may be important in improving long-term engagement. Low retention rate might also be due to an unguided self-help approach, and further engaging those who need self-monitoring remains challenging.

Another issue with attrition was that definitions varied significantly. While some studies reported users who only logged in a single time as *dropouts*, others expanded the definition to include those who only used an app once or twice. For example, the RCT with the lowest dropout rate overall included patients who only sent a single report through the app during the entire follow-up time, which did not indicate sustained, long-term use [29]. While these users may have been nondropouts by the definitions used in that study, they had received significantly lower benefits from the intervention, and would likely be considered dropouts had the analysis been less broad.

## Discussion

### Principal Findings

Attrition in app-based interventions is an important and yet underresearched element. For these interventions to work, it is a necessary component that people use and continue to use the app; however, there appears to be evidence that this is not always the case. In this systematic review and meta-analysis, the pooled estimate of dropout rates was 43%, with higher rates seen in real-world research and lower rates in highly supported RCTs. This may indicate a very serious underlying issue, as high dropout rates in these interventions will limit their use and uptake in health care across a range of chronic diseases.

While dropout rates in RCTs were notably lower than in observational trials, it is worth noting that attrition was often defined differently in this research. RCTs tended to describe all participants as users of the app unless they had ceased using it entirely; while this is in line with best-practice intention-to-treat analyses, it also presents an important limitation with the pooled results above. Including people in analyses who have been randomized is laudable; however, this also obscures the fact that large proportions of people, even in these randomized trials with detailed patient support and follow-up, barely used the app, if at all. This is worrying, because it implies that even with very high levels of support, apps are not an intervention with substantial staying power for people with various chronic diseases. It is also worth recognizing that combining estimates for both observational and RCT studies may have some drawbacks, given the differences in study design and participant retention. However, the difference in reported dropout rates was not significantly different, with only a small difference in point estimates and overlapping confidence intervals. This is likely because the main divergence was in definition of attrition, rather than the specifics of the study per se. As reported above, even among RCTs with low rates of dropout, there may have been a number of studies that would meet the looser definitions of *attrition* used in some observational research.

It was also concerning that there does not appear to have been much examination of the reasons behind this attrition in many studies. Few studies attempted to explain why people dropped out, with this being attributed to health literacy, age, and education, but it is unlikely that these are the only factors that would be related to attrition in the use of apps. For example, as mentioned in the results, people whose health saw greater improvements were more likely in some studies to keep using the app. It is likely that there are a range of unidentified issues that could potentially be targeted to ameliorate this problem, but thus far there has been little recognition of the issue formally, which may have limited the research that has been done to remedy the situation. Many studies do not even address the possibility that people dropping out of an app-based intervention at alarming rates could be an issue for the intervention’s adoption at scale, nor do they address the issue that this could cause in terms of aggravating health inequities depending on the reasons for dropout. This is especially concerning when considering that age and social status are likely to be barriers to app access—as some included studies hinted—which may further compound the issues caused by selection bias of those who use apps in the first place. If younger, healthier people are more likely to use apps overall, which is often the case [32], and are then more likely to use them long term, the apps may be less useful for the very populations that we most want them to help.

This is a common theme among trials, in which attrition or nonusage is barely addressed, or only given very surface-level appraisal. If there is a significant difference in the primary outcome between the intervention and control groups, there is a general attitude that the attrition is unimportant; this appears to be fairly common in RCTs, and may be because the aim of this research is specifically to evaluate the app in an intention-to-treat framework [29,30]. However, there are clear drawbacks to this, not least that we may be seeing a large underestimate in the literature of the efficacy of app-based interventions, caused by their generally low use in the populations who have been studied.

There are a number of very important limitations to this research. Firstly, the estimates produced are certainly not comprehensive. Many studies (N>30) that fit all of the inclusion criteria failed to report dropout or attrition in a way that could be extracted. Given the number of trials on app-based interventions, it was not considered feasible to follow up with every author group that had these figures, but it is worth noting that this best guess represents a relatively small number of trials within the total pool of potential evidence.

There is also the issue with heterogeneity. Given the nature of the included studies, it is not surprising to find very significant levels of heterogeneity statistically, but it is concerning for the meta-analysis as a reasonable estimate of attrition. These studies were conducted across different disease states, with highly variable interventions; the fact that they all included an app is a thin bond that did not overcome the vast differences that they had between them.

It is also worth noting that the research in this space is quickly evolving. We found no published research to be included before 2010, very little in the years leading up to 2015, and then an explosion of studies in the years after. It is likely that redoing this meta-analysis in 2025 will yield a much more reliable estimate of the figures. This may also allow for analyzing by disease state, which could prove to be a more accurate estimation of the rate of attrition.

There are a number of theories that might pertain to attrition in app-based interventions, with several different focuses. One area that could help inform attrition research in the future is behavioral theory, perhaps by examining the sociocognitive aspects of people who do and do not drop out of app-based studies. Integrated behavioral theories might also be useful in examining the relationship between social factors and the behaviors they cause, to come to an understanding of the process by which people decide to use or discontinue using apps.

This would ideally tie in to an examination of the broader social and demographic drivers of attrition. While few of these drivers have been identified in research so far—age, in particular—there remains a large evidential gap pertaining to how society influences behavior to prevent people from using app-based interventions. Future research should combine these two theoretical approaches to define the background reasons for attrition, so that interventions can be designed to minimize it.

Aside from the estimates of app attrition, there are some important implications of this research. Future studies looking at app-based interventions should include attrition as a secondary endpoint and develop methods to prevent it if possible. One important aspect would be to develop a standard measure of minimum use in app-based interventions; a reasonable example is the one used in some of the included trials of one or fewer log-ins to the app in any given period of time (ie, one log-in per month). Lower use than this basic threshold could then be considered *attrition* for the purposes of research studies. As well, there should be trials looking at ways to reduce the rate of dropouts, as well as the potential inequity in the rate of attrition, in app-based interventions. Without such research, we have no way of knowing if apps can be effective in the general population.

### Conclusions

This systematic review and meta-analysis found that the pooled estimate for dropout rates in trials of app-based interventions for chronic diseases was 43% over a variety of timelines, with the length of time having little impact on the rate of dropout. Attrition was higher in observational *real-world* studies, with randomized clinical research seeing less than a third of patients drop out before the trials were completed. However, findings were limited by high heterogeneity and the lack of reporting in many trials on attrition rates. Future research should focus on how often patients drop out and examine reasons why, so that this important issue can be addressed in app-based interventions for chronic disease.

## Abbreviations

CENTRAL: Central Register of Controlled Trials
MEDLINE: Medical Literature Analysis and Retrieval System Online
mHealth: mobile heath
RCT: randomized controlled trial
